# Knowing a synapse when you see one

**DOI:** 10.3389/fnana.2015.00100

**Published:** 2015-07-28

**Authors:** Alain Burette, Forrest Collman, Kristina D. Micheva, Stephen J. Smith, Richard J. Weinberg

**Affiliations:** ^1^Department of Cell Biology and Physiology, University of North Carolina at Chapel HillChapel Hill, NC, USA; ^2^Allen Institute for Brain ScienceSeattle, WA, USA; ^3^Department of Molecular and Cellular Physiology, Stanford UniversityStanford, CA, USA

**Keywords:** synapse, network, cortex, microscopy, array tomography, electron microscopy, fluorescence

## Abstract

Recent years have seen a rapidly growing recognition of the complexity and diversity of the myriad individual synaptic connections that define brain synaptic networks. It has also become increasingly apparent that the synapses themselves are a major key to understanding the development, function and adaptability of those synaptic networks. In spite of this growing appreciation, the molecular, structural and functional characteristics of individual synapses and the patterning of their diverse characteristics across functional networks have largely eluded quantitative study with available imaging technologies. Here we offer an overview of new computational imaging methods that promise to bring single-synapse analysis of synaptic networks to the fore. We focus especially on the challenges and opportunities associated with quantitative detection of individual synapses and with measuring individual synapses across network scale populations in mammalian brain.

## Introduction

*What we see depends mainly on what we look for*.

—John Lubbock (1892; Lord Avebury)

The individual synaptic contact is the fundamental element of all synaptic network signaling, including the human neocortical signaling that allows the reader to parse this sentence. We know that the myriad synapses of mammalian CNS are richly complex and diverse in structure, composition, and function, but so far our knowledge about the patterning of synapse diversity over the cubic millimeter scale of cortical local networks is very limited. With growing recognition that the processing, storage, and retrieval of information by CNS networks must be rooted fundamentally in dynamic patterns of individual synaptic weights, the shortage of information on synapse-level population diversity poses a major obstacle to understanding CNS synaptic network function. This shortage has resulted largely from technical barriers to identifying and measuring entities as small, complex, numerous and densely packed as the synapses in mammalian brain. Fortunately, methodology advances are now opening new avenues toward the analysis of large and diverse synapse populations with single-synapse resolution. Here we consider some of these new single-synapse analysis methods and how they are likely to advance our understanding of brain mechanisms and function.

## What is a Synapse?

Sherrington’s prescient term (Bennett, 1999; López-Muñoz and Alamo, 2009) did not become an anatomical reality until the 1950’s, when electron microscopists demonstrated the characteristic structure of the synapse (Robertson, 1953; Peters et al., 1976). Notwithstanding differences between mammals and invertebrates, and between the neuromuscular junction and the CNS, the basic morphology of the synapse—a vesicle-rich axon terminal making a specialized electron-dense adhesive contact onto its postsynaptic target—is unmistakable and now represented in every neuroscience textbook. Ultrastructural study can distinguish two main types of synapses, generally corresponding to excitatory (“asymmetric, ” or Gray Type I), and inhibitory (“symmetric, ” or Gray Type II; Gray, 1959; Colonnier, 1968). This dichotomy between excitatory and inhibitory synapses has held up well, notwithstanding considerable heterogeneity among each of these types, as well as the presence of less common synapses that do not fit into a binary structure.

Electrophysiological study can also define synaptic connectivity, by measuring an evoked postsynaptic potential following the induction of a presynaptic spike. Electrophysiology can distinguish different functional types of synapses, characterizing their sign, time course, and patterns of short term and long-term plasticity. However, physiological detection of synapses faces an array of technical problems, including uncertainties as to dendritic loci of synaptic contact sites, and signal distortion consequent to electrical distance between the recording site and synaptic locus. Some synapses have been shown to be “silent, ” and some do not produce conductance change unless some modulatory condition is fulfilled (Millar et al., 1976; Kerchner and Nicoll, 2008; Crawford and Mennerick, 2012). Others produce no conductance changes at all, but act exclusively via “metabotropic” chemical signaling mechanisms.

More broadly, it is now clear that synapse populations of the mammalian cortex are extremely diverse in composition, structure and function (O’Rourke et al., 2012). This heterogeneity goes far beyond traditional excitatory/inhibitory or neurotransmitter categories and poses an experimental challenge best answered by single-synapse analysis. Unfortunately, the small size and dense packing of neocortical synapses pose formidable obstacles to single-synapse analysis. Electrophysiology and electron microscopy have provided the foundations of our modern understanding of synaptic mechanisms, but neither modality in its traditional form is suitable for the analysis of diverse individual synapses at the scale needed to build a mechanistic understanding of CNS network function. Even “simple” neocortical networks such as the well-studied rodent whisker column comprise hundreds of millions of densely-packed synapses, precluding the satisfactory application of traditional labor-intensive approaches to single-synapse analysis. Emerging methods of computational microscopy (Burns et al., 2013) are now poised to advance single-synapse imaging and measurement to the necessary scale, so careful thought is now due to questions about what we should be looking for, and what we need to measure once we see it.

## Fluorescence Microscopy

Fluorescence microscopy is a core tool of synapse biology, invaluable for live cell, histochemical and multimodal applications including optical study of synapses in the awake behaving animal. Unfortunately, the limited resolution of conventional light microscopy poses problems for the study of synapses. This problem is manageable in cultures (which contain sparse neuropil confined to two dimensions; Craig et al., 1993) but can severely compromise use in the intact brain, unless sparse labeling methods are used. We have learned a lot about the function of individual synapses in culture, and now many new genetic approaches to both targeted and shotgun sparse labeling in brain tissue are now being put to excellent use.

Confocal microscopy has been used very effectively to measure individual synapses in cortical tissues (Dumitriu et al., 2011; Schoonover et al., 2014), but suffers resolution and depth limitations that have restricted quantitative application at the local network scale. New super-resolution optical microscopy approaches yield outstanding images of single-synapse molecular architecture (Ji et al., 2008; Eggeling et al., 2015), but again have so far not been extended to the scale of a cortical local network. A variety of new tissue clearing methods (Miyawaki, 2015) are raising hopes for network-scale imaging and single-synapse resolution, especially if appropriate 3D super-resolution microscopy modalities can be developed. These technologies have already provided exciting new data on synapse properties and offer great prospect for future rapid improvements in neural tissue imaging. Another promising new approach to effective super-resolution histology is based on isotropic expansion of tissue labels by an expanding gel matrix (Chen et al., 2015).

## Optophysiology

Recent advances in fluorescence imaging methods and reagents have made possible the non-invasive measurement of function at the single-synapse level in intact cortical neuropil. Calcium-sensitive fluorescent molecules, both synthetic and protein based, allow the measurement of calcium influx at individual spines and boutons. Although originally applied for *in vitro* studies, recent progress has been made towards applying them *in vivo*, so that the functional properties of synapses can be mapped in the context of animal behavior (Rochefort and Konnerth, 2012). Voltage-sensitive molecules (both synthetic and protein-based) are the most direct probes of membrane potential at individual synapses, but have so far seen rather limited use (Palmer and Stuart, 2009; Canepari et al., 2010; Maclaurin et al., 2013; Hochbaum et al., 2014; St-Pierre et al., 2014). Fluorescence Lifetime Microscopy (FLIM) and Fluorescence Resonance Transfer (FRET) have also provided fascinating new glimpses of molecular signaling at individual cortical synapses (Yasuda, 2006).

## Electron Microscopy

**Figure 1:**
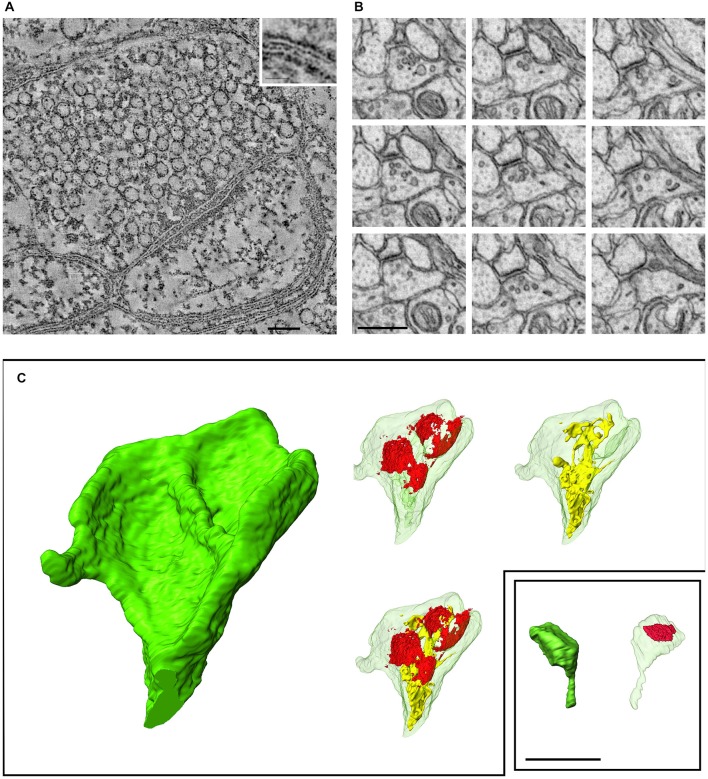
**Recent developments in electron microscopic imaging of synapses. (A)** Electron tomography (cerebral cortex), a 2.2 nm-thick computed tomographic slice through the center of a synapse. The tomographic section was reconstructed from a dual-axis tilt series of images (from −65° to + 65°, with 2° increments) from cerebral cortex. Inset (enlargement of boxed region) shows the plasma membrane more clearly; the external surfaces of the lipid bilayer are lined with electron-dense particles (Burette et al., 2012). **(B)** Focused Ion Beam Scanning Electron Microscopes (FIBSEM) illustrates a series of ultrathin sections through a small synapse in nucleus accumbens (25 nm spacing between sections); such a small synapse would be likely to escape detection with standard serial-section TEM. **(C)** Three-dimensional reconstruction of two spines (ventral striatum) from a FIBSEM stack (red shows synaptic apposition; endomembranes shown in yellow); the two spines are shown to the same scale. Scale bars: **(A)**, 100 nm; **(B**,**C)**, 500 nm.

Notwithstanding remarkable recent progress in photon-based imaging, spatial resolution remains an obstacle for fluorescence-based study of synapses. Can electrons help? Using a technology already mature 30 years ago, the transmission electron microscope (TEM) routinely provides nm-level resolution in the X-Y axis, though technical constraints preclude its use on live tissue. While Z-axis resolution is more limited (~50 nm), this is ample to allow crisp visualization of even the smallest synapses. However, several problems can make it difficult to identify a synapse even with TEM, including unfortunate plane of section (addressable by study of serial thin sections), the perhaps surprising abundance of tiny synapses (Figure 1), and synaptic immaturity (bearing in mind that synaptogenesis is present even in the adult brain).

Beyond the problem of synapse recognition, classical TEM technology is labor-intensive, especially as extended by serial-sectioning into 3D, and therefore poorly suited for large-scale study of synaptic neuropil. Stereological approaches to sampling can reduce the magnitude of the task (Vrensen and de Groot, 1973; DeFelipe et al., 1999; Witgen et al., 2006), but require careful experimental design and yield limited results. Several recent developments promise to make EM of bulk tissue more feasible (Knott and Genoud, 2013; Kubota, 2015), including “industrialization” of specimen preparation and image acquisition (Briggman and Bock, 2012), and the introduction of novel high-throughput scanning EM-based techniques, including the automatic tape-collecting ultramicrotome (Hayworth et al., 2014), serial block-face microscopy (Denk and Horstmann, 2004), and focused ion-beam methods (Knott et al., 2008). These methods were originally developed for connectomics, but Focused Ion Beam Scanning Electron Microscopes (FIBSEM) has now been adapted to provide exquisite visualization and precise measurement of synapses and on large synapse populations (Figure 1; Merchán-Pérez et al., 2009). Unfortunately, these tools are poorly suited for studies of synapse molecular heterogeneity.

## Combining Optical and Electron Microscopy Methodologies

Because the strengths of optical and electron microscopy are complementary, putting these two methodologies together for large-scale studies of the brain has long been a coveted goal. While progress has been delayed by the often mutually-exclusive sample preparation requirements, recent advances are beginning to reveal the power of approaches that successfully incorporate large-scale optical and electron microscopy imaging, such as array tomography (AT) and automated TEM.

AT is an emerging technology well-suited to the challenges of assessing synaptic diversity quantitatively on cortical local network scale. The underlying technology is conceptually simple: thin (~70 nm) serial sections cut from plastic-embedded pieces of fixed brain are collected onto coverslip arrays, yielding Z-axis resolution at least 10 times higher than provided by confocal microscopy. The arrays are immunostained using antibodies against three to four different substances of interest, and images are collected with immunofluorescence. Because the method relies on postembedding immunostaining, it minimizes an otherwise troublesome problem: using standard “pre-embedding” techniques, antibody access is problematic at protein-dense regions like synapses; this may require the use of proteolytic agents or very weak fixation, which in turn can cause problems with structural artifacts and disruption (Fukaya and Watanabe, 2000; Burette et al., 2002). The immunostains are then eluted under denaturing conditions, the arrays are restained with new antibodies, and imaged again, over multiple cycles. The resulting data are computationally assembled into Z-axis stacks.

**Figure 2:**
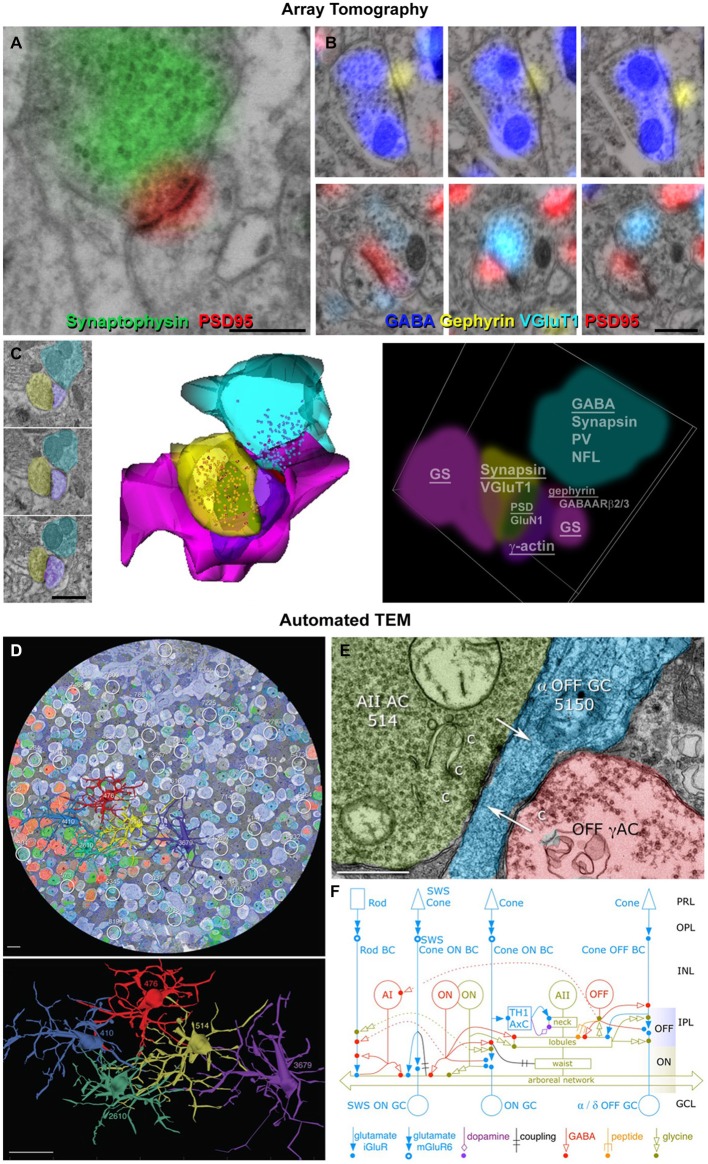
**Enhanced view of synapses using optical and electron microscopy. (A)** Conjugate array tomography (AT) view of the ultrastructural features of a synapse (presynaptic vesicles, postsynaptic density, surrounding glia) and typical synaptic molecular markers (synaptophysin in the presynaptic compartment and postsynaptic density protein 95, PSD95 on the postsynaptic density). Scale bar, 500 nm. **(B)** Serial sections from an inhibitory (top) and excitatory (bottom) synapses. Scale bar, 500 nm. **(C)** Conjugate AT allows for detailed ultrastructural and molecular characterization of synaptic arrangements. A dually innervated spine from the mouse somatosensory cortex was imaged on serial sections (left), reconstructed from SEM images (middle) and from light microscopic images probing for 17 different synaptic and cytoskeletal markers (right). The underlined markers were used to reconstruct the different structures. Scale bar, 1 μm. **(D)** Automated transmission electron microscope (ATEM) view of the retinal connectome including synapse parent neurons, molecular markers and ultrastructure. A retinal slice overlaid with five different molecular markers and five reconstructed AII amacrine cells. Scale bars, 10 μm (up), 20 μm (bottom). **(E)** A synaptic contact made by cell 514 onto an OFF ganglion cell. Scale bar, 500 nm. **(F)** The new richer network description of AII amacrine cells obtained using ATEM. Reproduced with permission from Marc et al. (2014).

Current techniques allow semi-automatic collection of high-dimensional proteometric data (>25 antibodies) on sizable (1000 × 200 × 50 μm^3^) chunks of brain, at a resolution of 200 × 200 × 70 nm, which can be further improved to 100 × 100 × 70 nm using deconvolution (Wang and Smith, 2012; Figure 2). Much larger volumes (1 × 0.83 × 0.21 mm^3^) have been successfully imaged after a single cycle of immunostaining with three different antibodies to identify thalamocortical synapses (Rah et al., 2013). AT arrays can then be imaged with scanning EM (“conjugate AT”), and the resulting images co-registered with single synapse precision (Oberti et al., 2011; Collman et al., 2015). Using conjugate AT, we recently confirmed that at least 90% of excitatory synapses are correctly identified over a wide range of sizes with fluorescence-based AT (Figure 2). Synapse identification could be further improved by combining AT with mGRASP (Kim et al., 2015), an approach likely to accelerate network analysis in the near future. The prospects look excellent for scaling up AT methods to acquire fluorescence images economically at the local network scale while retaining the opportunity to image selectively with Field emission scanning electron microscopy (FESEM) as needed for increased resolution and visualization of axonal, dendritic and synaptic membrane structures. Combinations of optophysiological fluorescence and AT imaging may prove an ideal match to the challenges of discovering the diverse molecular counterparts of diverse function at the level of individual synapses and doing so on the mm^3^ volume scale of complete local networks.

Another new technology, called Automated TEM (ATEM), has already combined optical and electron microscopy to study of the retina at network scale. Like AT, ATEM uses serial ultrathin sections of plastic-embedded chemically fixed tissue, but performs immunohistochemistry on separate single sections intercalated within the long series of sections that are viewed by TEM. Registration of the optically imaged immunostained sections with the EM sections allows the molecular composition of cells to be established; their processes are then traced and synapses ultrastructurally identified on the TEM sections. Up to 11 different antibody labels have been used in this method, mostly against small molecules, such as GABA, glutamate and other neurotransmitters. Importantly, prior *in vivo* activity can be probed using the excitation marker 1-amino-4-guanidobutane (AGB), a channel-permeant organic cation whose presence can be subsequently detected in the fixed tissue using an antibody (Marc et al., 2005). Further advantages of this method are the very high resolution enabled by the use of TEM (down to 0.5 nm lateral resolution), and the automated nature of TEM image acquisition that has allowed the imaging and full reconstruction of a retinal circular segment with a diameter of 0.22 mm and approximate thickness of 0.03 mm. ATEM allows the collection of terabyte to petabyte image volumes which, similarly to AT, require new image processing, assembly, navigation and analysis algorithms, as well as new interpretive frameworks. Ongoing exploration of the acquired retinal volume has uncovered much greater complexity of the retinal synaptic network than previously acknowledged and recognized, and has demonstrated the existence of a number of new connection motifs and functions (Marc et al., 2013). Some of these newly-described contact architectures are now challenging the classical ultrastructural definition of a chemical synapse that is still used as the ultimate criterion for synapse identification.

## So, Really, Is this a Synapse?

Several decades ago, the ultrastructural definition of a chemical synapse was simple and precise. As emphasized by Alan Peters and Sanford Palay, “only the clear presence of all the principal features of the synaptic junction can verify the presence of a synapse: the presynaptic vesicles in characteristic clusters, the presynaptic densities, the synaptic cleft, and the postsynaptic densities” (Peters and Palay, 1996). Many EM studies further restricted this definition by requiring the presence of at least three presynaptic vesicles (Beaulieu and Colonnier, 1985). The goal at the time was to understand the synapse as a possibly invariant unit, and it only made good sense to focus on what one was certain to be a synapse.

Now, with goals of understanding network architectures in mind, the classical criterion for recognizing a synapse may be much too narrow. Synapses missed by standard electron microscopy because they are too small or immature to meet traditional criteria may nonetheless exist in such large number as to heavily impact network function (Buzsáki and Mizuseki, 2014). Small and immature synapses may also play critical roles in network plasticity and homeostasis that endow them with special importance. For instance, although small synapses may transmit only weakly or rarely, they may be the primary substrate of new memory formation (Fu et al., 2012). To truly understand the brain’s synaptic networks, comprehensive new criteria may be needed to incorporate the abundant small but ill-defined synapses, as well as other somewhat atypical contact architectures. As a result of the technological advances outlined above, criteria for synapse identification may now include molecular, physiological, and ultrastructural characteristics. For these criteria to be useful in practice, they may need to overlap heavily, allowing the choice of different subsets of criteria to recognize a synapse. For example, a synapse sectioned *en face* will not show a clear postsynaptic density, but the presence of postsynaptic density protein 95 (PSD-95) or another synaptic scaffold protein might be sufficient as an affirmative criterion. While small, newly formed synapses may lack classical ultrastructural features and detectable concentrations of PSD proteins, evidence of synaptic signaling provided by optical physiology might confirm functional synaptic identity.

## More Challenges and Opportunities Ahead

To understand the mechanisms of human neocortical information processing, we must grapple with intricacies of a machine that packs nearly a billion miraculous computing machines called “synapses” into each cubic millimeter of wet volume. Each synapse represents ~10^5^ signaling proteins acting in some as yet unknown kind of concert. And each synapse is different. Indeed, a strong case can be made that our skills, memories, and emotional predilections are encoded primarily in our personal patterns of synaptic diversity, that is, in the differences amongst our synapses!

We need more powerful and economical means of measuring synaptic diversity patterns with single-synapse granularity, and to be able to relate those patterns, perhaps via modeling, to network function. These tools need to operate reliably and quantitatively over local network scale—volumes of approximately a cubic millimeter in mammalian cortex—and over a wide range of synapse types and sizes. We need to know how the synapse inherits its molecules and functional characteristics from its parent neurons, and how molecular and structural characteristics of the synapse predict and dictate synaptic function. Moreover, distinctive adhesion proteins concentrated at synapses likely encode much information about the identity, morphology and anatomic loci of the parent neurons. Tools like those we have mentioned here are likely soon to be reading such molecular codes.

Quantitative reliability of all the single-synapse analysis tools we have mentioned rely upon a person or (increasingly) an algorithm that *knows a synapse when it sees one*. The synapse recognition process must function predictably across a daunting range of anatomical scales and synapse sizes and forms, and deal with the many measurement complications discussed above. It still poses a great challenge to experimental neuroscience. The new technologies we have discussed here nonetheless inspire optimism that single-synapse analysis tools suitable for wide use in neuroscience will soon emerge.

## Conflict of Interest Statement

SJS and KDM have founder’s equity interests Aratome, LLC (Menlo Park, CA), an enterprise that produces array tomography materials and services. SJS and KDM are also listed as inventors on two US patents regarding array tomography methods that have been issued to Stanford University.
